# Sequence-based modeling of low-affinity transcription factor–DNA binding through deep learning

**DOI:** 10.1093/nargab/lqag027

**Published:** 2026-03-05

**Authors:** Yingfei Wang, Jinsen Li, Tsu-Pei Chiu, Beibei Xin, Remo Rohs

**Affiliations:** Department of Quantitative and Computational Biology, University of Southern California, Los Angeles, CA 90089, United States; Department of Quantitative and Computational Biology, University of Southern California, Los Angeles, CA 90089, United States; Department of Quantitative and Computational Biology, University of Southern California, Los Angeles, CA 90089, United States; State Key Laboratory of Maize Bio-Breeding, Department of Plant Genetics and Breeding, China Agricultural University, Beijing 100193, China; Department of Quantitative and Computational Biology, University of Southern California, Los Angeles, CA 90089, United States; Department of Chemistry, University of Southern California, Los Angeles, CA 90089, United States; Department of Physics & Astronomy, University of Southern California, Los Angeles, CA 90089, United States; Thomas Lord Department of Computer Science, University of Southern California, Los Angeles, CA 90089, United States; Division of Medical Oncology, Department of Medicine, University of Southern California, Los Angeles, CA 90033, United States; Alfred E. Mann Department of Biomedical Engineering, University of Southern California, Los Angeles, CA 90089, United States

## Abstract

Multiple layers of molecular determinants and mechanisms affect binding specificity between transcription factors (TFs) and DNA. DNA sequence-based deep learning models using convolutional neural networks (CNNs) and self-attention (SA) transformers have improved modeling accuracy and advanced our understanding of TF–DNA binding specificity through network interpretation. However, the systematic evaluation of various strategies for handling DNA sequence orientations in deep learning models—and their interpretation—remains underexplored, especially in the context of learning low-affinity binding site specificity. Using SELEX-seq data for eight Exd-Hox heterodimers in *Drosophila*, we compared canonical models with data augmentation and reverse-complement weight-sharing models. We found that reverse-complement weight-sharing CNN models and SA models trained with augmented data with reverse complements outperformed other approaches in modeling binding specificity. In this work, we evaluated several interpretation methods, including Gradient*input, DeconvNet, DeepLIFT, and *in silico* mutagenesis (ISM). Compared to other interpretation methods, ISM was less sensitive to model hyperparameter settings. In this work, we identified Exd-Ubx binding at low-affinity sites and suggested possible biophysical mechanisms. The findings of this study will be relevant for studying the functional role of low-affinity TF binding in gene regulatory mechanisms with possible implications on TF–DNA binding specificity guided protein design.

## Graphical Abstract

**Graphical Abstract:**
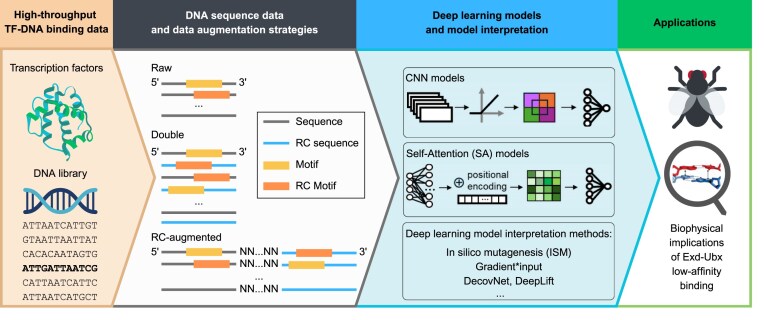

## Introduction

Transcription factor (TF) binding on specific DNA target sites plays a crucial role in regulating gene expression. Unraveling the mechanisms that govern TF–DNA binding specificity is necessary to explain the precise gene expression patterns that further determine cell fate [1, 2] and developmental patterning [3] (i.e. Hox proteins in *Drosophila* [4]). TFs typically bind to DNA motifs with 6 to 20 base pairs (bp) in length. These motifs can appear numerous times in a genome, yet fewer than 1% of these putative TF binding sites (TFBSs) are functional in a cellular environment *in vivo* [5–7]. Our current understanding of TF–DNA binding specificity covers *in vitro* biophysical determinants (i.e. DNA sequence context, cofactor/cooperativity, and local DNA shape profiles) [8–10] and *in vivo* genome-wide determinants (i.e. chromatin accessibility, epigenetic marks, and 3D genome structure) [11, 12].

Fortunately, comprehensive data have been generated through high-throughput sequencing techniques for TF–DNA binding (SELEX-seq for *in vitro* [13] and ChIP-seq for *in vivo* TF–DNA binding [14]) and genome-wide profiles of epigenetic binding specificity determinants (DNase-seq/ATAC-seq for chromatin accessibility [15, 16], ChIP-seq for histone modification patterns [17], and bisulfite sequencing for DNA methylation). With these data, investigating the importance of a certain binding specificity determinant or interplay among multiple determinants has become both timely and feasible for understanding TF–DNA binding mechanisms. Because complex interactions occur in the nucleus (e.g. nucleosome positioning, histone modifications, DNA modifications, cooperative binding, etc.), modeling TF–DNA binding mechanisms through *in vitro* datasets provides a good entry point.

The simplest model to understand what DNA sequence a TF prefers is to use a position weight matrix (PWM) [18]. However, a PWM is insufficient to model the complex TF–DNA readout mechanism because it does not consider interdependency between nucleotide positions [19]. With the availability of more high-throughput datasets [20, 21], modeling TF–DNA binding using machine learning models has become a common approach [22]. Machine learning models developed to predict TF–DNA binding using either DNA sequence, DNA shape features, or both can usually be fitted into two major categories: explainable methods [e.g. L2-regularized multiple linear regression (MLR)] and “black-box” methods (e.g. deep learning models).

Traditional explainable methods usually start with alignment of sequences to the canonical motif [23]. Sequence alignment allows traditional machine learning methods to learn the importance of position-specific nucleotides within or around the TFBS, using features such as DNA sequence and shape [24]. However, sequence alignment sacrifices substantial information by considering only the most likely cognate TFBSs. Low-affinity sequences are usually discarded during this process. This problem can be partially handled through *de novo* motif discovery models that utilize complete sets of raw sequences and learn a PWM or PWM-like matrix [25, 26]. Most such models assume independent biophysical contributions of each nucleotide position to TF–DNA binding affinities; thus, they are incapable of capturing higher-order dependencies of nucleotides within TFBSs. Although *k*-mer-based biophysical models consider higher-order dependencies [27, 28], this approach does not guarantee higher performance in modeling TF–DNA binding affinity, nor does it guarantee that interactions between non-adjacent nucleotides are captured. Therefore, with TF–DNA binding data, each of these methods has limitations in studying the importance of individual nucleotides in TFBSs, let alone in studying the interplay of different determinants of TF–DNA binding.

Deep learning models, especially those using CNNs, have become increasingly popular for predicting TF–DNA binding [29, 30], largely because they are alignment-free methods that can take raw sequences as input, ensuring that no reads are discarded. They capture non-linear dependencies between nucleotides within TFBSs, excel in modeling binding accurately, and have high computational scalability. Despite their superiority in many aspects, deep learning models have limitations in interpretability. Methods have been proposed to improve interpretability through gradient-based backpropagation methods [29, 30], DeepLIFT [31], or *in silico* mutagenesis (ISM) [32, 33], and previous work has applied these methods to study TF–DNA binding [34]. Alternatively, self-attention (SA)-based deep learning models can offer a powerful and interpretable alternative approach for predicting TF–DNA binding. The attention mechanism inherently highlights key sequence motifs and interactions, providing insights into the sequence determinants of TF binding affinity. SA models have proven useful for multiple tasks in computational biology, including predicting the DNA binding specificity of proteins [35–37]. Yet no systematic analysis has been performed to identify the method of choice in achieving the most robust interpretability for the task of predicting TF–DNA binding.

Through sequence-level interpretability, deep learning models may provide additional insights on low-affinity TF binding. Studies investigating the binding specificity of low-affinity TFBSs have gained increasing attention due to their critical role in fine-tuning gene regulation [38, 39]. Unlike high-affinity binding sites, which strongly attract TFs and are typically well-characterized, low-affinity sites bind TFs more weakly and are often poorly predicted by most PWM models [40, 41]. By utilizing different interpretation methods, we asked the question if deep learning models can provide more accurate quantification and mechanistic understanding of TF recognition across a broader range of binding affinities.

In this work, we developed a series of deep-learning models to study the binding mechanisms of TFs on unaligned SELEX-seq data for eight Exd-Hox heterodimers. We reviewed different strategies for handling reverse-complement strands for CNN models and showed how they can be used to improve model robustness. In addition to CNN models, we evaluated SA models to see if the attention mechanism improves TF–DNA binding prediction accuracy and interpretability. Next, we studied the robustness of different network interpretation methods to obtain unit-resolution importance matrices. In a case study, we recovered phenotypic evidence of Exd-Ubx binding at low-affinity TFBSs within the *shavenbaby* (*svb*) enhancer in *Drosophila* to achieve binding specificity [42]. Our study provides a systematic approach to building sensitive and consistent deep learning models, together with robust interpretation methods, to explain phenotypic evidence caused by genetic variation and cell type-specific binding events.

## Materials and methods

### SELEX-seq and input data augmentation methods

We collected round-3 SELEX-seq data from dataset GSE65073, generated by Abe *et al.* [43]. We used the 14-mer data for modeling, following the same procedure as described in Abe *et al.* [43]. Specifically, for each Exd-Hox heterodimer, the dataset contains a large number of 14-bp DNA sequences and their corresponding relative binding affinity, with values between 0 and 1. A summary of the sample size for each dataset is provided in Supplementary Table S1. We refer to these datasets as “raw data” or “SELEX-seq data.” We refer to the 14-bp DNA sequences in these datasets as “raw sequences” or “SELEX-seq sequences.”

**Figure 1.**
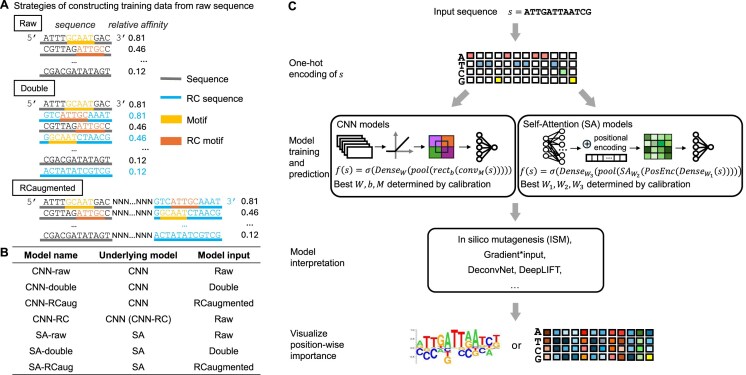
Strategies for handling input data and evaluating models. (**A**) Strategies of constructing training dataset from sequences and relative affinity in SELEX-seq data. (**B**) Summary table of strategies used by seven models. (**C**) Workflow of model training and interpretation for convolutional neural networks (CNNs) and self-attention (SA) models.

When training models, raw data are often preprocessed by data augmentation techniques. We refer to the dataset used for model training as “input data.” In this work, we studied different methods of DNA-specific data augmentation for constructing the input data: i.e. “Raw,” “Double,” and “RC-augmented.” The “Raw” approach was the baseline, in which no data augmentation was performed, and the raw SELEX-seq sequences and their relative binding affinity were used directly as input data for models. In the “Double” approach, we added the reverse-complement sequence of each raw SELEX-seq sequence into the dataset and assigned it the same relative binding affinity as in the original SELEX-seq sequence. After the data augmentation step, input data for model training had twice the number of sequences as in the SELEX-seq data. The “RC-augmented” approach involved modification of the sequences. We extended all SELEX-seq sequences by adding the reverse-complement sequence and padding 10 Ns in between. Input data consisted of 38-bp DNA sequences and their relative binding affinity (Fig. 1A and B).

### Aligned datasets and MLR with L2 regularization

To build an MLR model, sequences need to be aligned. First, we constructed the “Aligned” dataset by adopting the approach in Abe *et al.* [43]. We aligned all sequences in the raw SELEX-seq data based on the Exd-Hox heterodimer motif TGAYNNAY. Sequences that do not contain the motif or contain more than one core motif were not included in the analysis. In a second approach, we constructed a dataset using Top-Down Crawl [23], a motif-free alignment method aiming to remove as few reads as possible. We call this dataset “TDC-aligned.” Top-Down Crawl does not rely on regular expression or reduced sequence representation such as PWM-based approaches and keeps >98% of the raw data.

The aligned sequences are then one-hot encoded and used as the predictor $X$. The L2-MLR model fits $\hat{y} = Xw$ by minimizing the loss function:


\begin{eqnarray*}
L = \Vert y - Xw\Vert_2^2 + \lambda \Vert w\Vert_2^2,
\end{eqnarray*}

where the second term penalizes large weights. We determined the Ridge penalty coefficient λ with 10-fold cross validation.

### Model architecture and training process of CNN model

Our goal was to provide a systematic framework for model evaluation and interpretation, and to focus less on model performance. For this reason, we aimed to keep the model architecture simple and retained only crucial designs of each model type. For CNN-raw, CNN-RC-augmented, and CNN-double, we designed models $f( x )$ to predict the relative binding affinity of input sequence $input\_s$, using the following operation types:


\begin{eqnarray*}
f\left( {\textit{input}\_s} \right) = \sigma (\textit{Dens}{{e}_W}\left( {\textit{pool}\left( {rec{{t}_b}\left( {con{{v}_M}\left( {\textit{input}\_s} \right)} \right)} \right)} \right)).
\end{eqnarray*}

Convolutional layer ($con{{v}_M}$) used a set of motif scanners with parameters $M{\mathrm{\ }}$to scan a one-hot encoded matrix ($4 \times N$) of sequence $s$. Each scanner was a $4 \times m$ matrix. This step was followed by a rectification step, in which a shift $b$ was added to the output of each motif scanner, and *ReLU* was applied as an activation function. Afterward, we applied a max pooling layer with window size *p*, followed by a fully connected dense layer with a dropout design. Finally, a sigmoidal function was applied to generate the final prediction of the relative binding affinity, a value between 0 and 1. Formally, the aforementioned layers were defined as:


\begin{eqnarray*}
rec{{t}_b}(con{{v}_M}\left( {{{s}_k}} \right)) = \textit{ReLU}\left( {\mathop \sum \limits_{i = 1}^4 \mathop \sum \limits_{j = 0}^{m - 1} {{M}_{i,j}}{{s}_{i,j + k}} + {{b}_1}} \right)
\end{eqnarray*}

\begin{eqnarray*}
\textit{pool}\left( {{{x}_k}} \right) = \max \left( {\left\{ {{{x}_{kp}},\ {{x}_{kp + 1}},\ \cdots,\ {{x}_{kp + p - 1}}} \right\}} \right)
\end{eqnarray*}

\begin{eqnarray*}
\textit{Dens}{{e}_w}\left( x \right) = Wx + b
\end{eqnarray*}

\begin{eqnarray*}
\sigma \left( x \right) = \textit{sigmoid}\left( x \right) = \ \frac{1}{{1 + {{e}^{ - x}}}}.
\end{eqnarray*}

We defined the sum of the mean squared error (MSE) and scaled L1 and L2 penalizations on the convolution filter weights as the loss function:


\begin{eqnarray*}
L = \frac{1}{{\mathrm{N}}}\mathop \sum \limits_{i = 1}^N {{\left( {{{y}_i} - f\left( {{{x}_i}} \right)} \right)}^2} + {{\lambda }_1}\|M{{\|}_1} + {{\lambda }_2}\|M{{\|}_2^2},
\end{eqnarray*}

where ${{\lambda }_1}$ and ${{\lambda }_2}$ are weight penalization factors. We used the Adam gradient-based optimization algorithm [44] to update parameters, with a learning rate of ${{\lambda }_3}.$

Besides CNN-raw, CNN-RC-augmented, and CNN-double, we also implemented a special architecture described by Shrikumar *et al.* [45]. This was a reverse-complement weight-sharing CNN model (named here “CNN-RC”) that treated $s$ and $s^{\prime}$ equally by scanning $s^{\prime}$ through a “constrained scanner” rather than an independent scanner (Supplementary Fig. S1). The “constrained” scanner ensured that the reverse complement of a channel at index $i$ was present at index $3 - i$, the input channel for the reverse-complement residue. Moreover, a weighted sum layer following the pooling layer was added to learn a positional weight for each channel separately and to combine the positionally weighted channel output [45]. Therefore, for a given sequence $s$, the CNN-RC model computed a binding score $f( s )$ using the following five operation types:


\begin{eqnarray*}
f\left( s \right) = \textit{Dens}{{e}_{{{W}_2}}}(\textit{Weighted}\_su{{m}_{{{W}_1}}}\left( {\textit{pool}\left( {rec{{t}_b}\left( {con{{v}_M}\left( s \right)} \right)} \right)} \right) ).
\end{eqnarray*}

When training CNN models, we split the SELEX-seq data randomly into the training set (80%) and the test set (20%) and used ${{R}^2}$ as the test performance metric. We initiated the hyperparameter search by sampling nine sets of $({{\lambda }_1},{{\lambda }_2},{{\lambda }_3})$ (Supplementary Table S2), and used a three-fold cross-validation to determine the best hyperparameter set. We closely monitored the training process to ensure that this setup did not invoke overfitting. Finally, we applied the same architecture and hyperparameters to train models for all TFs, respectively.

### Model architecture and training process of SA models

For SA-raw, SA-RC-aug, and SA-double, we designed models $f( x )$ to predict the relative binding affinity of an input sequence $input\_s$ using the following operation types:


\begin{eqnarray*}
f\left( s \right) = \sigma \left\{ {\textit{Dens}{{e}_{{{w}_3}}}\left[ {\textit{pool}\left( {S{{A}_{{{w}_2}}}\left( {\textit{PosEnc}\left( {\textit{Dens}{{e}_{w1}}\left[ s \right]} \right)} \right)} \right)} \right]} \right\}.
\end{eqnarray*}

The first layer $Dens{{e}_{{{W}_1}}}$ mapped the one-hot encoded DNA sequence ($4 \times l$) into an embedding vector of higher dimension $( {{{d}_{\textit{model}}}\ \times l} )$. Next, we implemented the $PosEnc$ layer as used in classical transformer models (using the sin and cos functions), which pre-computed positional encoding and added it to the embedding vector, enabling the downstream SA layer to be aware of positions of residues in the sequence. Next, the SA layer $S{{A}_{{{W}_2}}}$ was applied to the positionally encoded vector, where ${{W}_2}$ was composed of ${{W}_V},\ {{W}_Q},\ {{W}_K}\epsilon {{R}^{{{d}_{\textit{model}}} \times {{d}_{\textit{model}}}}}$. A global average pooling layer was performed by averaging over the sequence dimension to generate a vector of size $( {{{d}_{\textit{model}}}\ \times 1} )$, representing aggregated information for the input sequence. Lastly, another *Dense* layer followed by a sigmoidal function was introduced to output a single value between 0 and 1 as the predicted relative binding affinity. Formally, the aforementioned layers were defined as:


\begin{eqnarray*}
h = \textit{Dens}{{e}_{{{W}_1}}}\left( s \right) = {{W}_1}s + {{b}_1}
\end{eqnarray*}

\begin{eqnarray*}
h^{\prime} = \textit{PosEnc}\left( h \right) = h + \textit{posenc}\left( h \right),
\end{eqnarray*}

where $posenc{{( h )}_{i,2k}} = {\mathrm{sin}}( {\frac{i}{{10{{{000}}^{\frac{{2k}}{{{{d}_{\textit{model}}}}}}}}}} ),{\mathrm{\ }}\textit{posenc}{{( h )}_{i,2k + 1}} = {\mathrm{cos}}( {\frac{i}{{10{{{000}}^{\frac{{2k}}{{{{d}_{\textit{model}}}}}}}}}} ),{\mathrm{\ }}i$ was the position index in the sequence, $k$ indexed the feature dimension, and ${{d}_{\textit{model}}}$ was the embedding size.


\begin{eqnarray*}
{{h}_{SA}} = SA\left( {h^{\prime}} \right) = \textit{softmax}\left( {\frac{{Q{{K}^T}}}{{\sqrt {{{d}_k}} }}} \right)V,
\end{eqnarray*}

where $Q = h^{\prime}{{W}_Q},{\mathrm{\ }}K = h^{\prime}{{W}_K},{\mathrm{\ }}V = h^{\prime}{{W}_V}$, and${\mathrm{\ }}{{d}_k} = \frac{{{{d}_{\textit{model}}}}}{{{{n}_{\textit{head}}}}}{\mathrm{\ }}$was the dimension of key vectors.


\begin{eqnarray*}
{{h}_{\textit{pool}}} = \frac{1}{l}\mathop \sum \limits_{i = 1}^l {{h}_{SA,i}}
\end{eqnarray*}

and $y = \sigma ( {{{W}_3}{{h}_{\textit{pool}}} + {{b}_3}}).$

For the loss function, we used the MSE metric:


\begin{eqnarray*}
{{L}_{MSE}} = \frac{1}{{\mathrm{N}}}\mathop \sum \limits_{i = 1}^N {{\left( {{{y}_i} - f\left( {{{x}_i}} \right)} \right)}^2}.
\end{eqnarray*}

When training the SA models, we split SELEX-seq data randomly into the training set (80%) and test set (20%) and used ${{R}^2}\ $as the test performance metric. For model architecture and training parameters, we first performed a grid search with varying values of learning rate, batch size, embedding dimension, numbers of SA layers, and attention heads (Supplementary Table S3). We roughly trained the model for 30 epochs and selected the most stable and best-performing training parameters to further investigate model architecture differences. We then thoroughly trained SA-raw models with varying numbers of attention layers and attention heads and selected the setup that almost always performed the best across all TFs (*nlayer *= 2 and *nhead *= 8) (Supplementary Table S4). We closely monitored the training process to ensure that this setup did not invoke overfitting. Finally, we applied the same architecture and training setups to train models for all TFs.

### Interpretation methods to obtain the unit-resolution importance logo

Interpretation methods vary in underlying principles and were implemented separately (Fig. 1C). DeepLIFT for CNN models was implemented according to its GitHub version v0.6.6.2-alpha. Gradient-based backpropagation methods were implemented through *Keras* built-in functions for CNN models (DeconvNet [30] and Gradient*input [31]), and through the *pytorch* built-in *autograd* method for SA models (Gradient*input). The SHAP framework for SA models was implemented with the *GradientExplainer* method using the *shap* package in Python [46, 47]. We reported two sets of SHAP results, using either all zeros or the training set as the background sequence.

ISM was implemented as follows. First, we computed the predicted binding affinity of each original sequence${\mathrm{\ }}f( s )$. Then, for every position ${{s}_i}$, we mutated it into one of the other possible nucleotides ${{b}_j}\epsilon \{ {A,T,C,G} \}$ as sequence $\hat{s}$. Second, we calculated $f( {\hat{s}} )$ and finally defined ${\mathrm{\Delta }}{{s}_{ij}} = ( {f( {\hat{s}} ) - f( s )} ) \cdot {\mathrm{max}}( {f( s ),\ f( {\hat{s}} )} )$. The second term in the product was used to override no binding and highlighted the magnitudes of change in strong binding. When interpreting the importance of a sequence of length larger than $l$ (e.g. the *svb* enhancer), we adopted a sliding window approach: at each position $i$, we report the model prediction using the subsequence from the ${{i}^{th}}$ and the ${{( {i + 13} )}^{th}}$ position.

For all above-mentioned methods, derived scores were in a matrix of size $4 \times l$, with each element representing the importance of each nucleotide at each position in predicting the binding affinity of the sequence $s$. For visualization purposes, only the importance level corresponding to the residue in sequence (e.g. the importance of adenine will be visualized at position 1 if the first nucleotide of $s$ is A) will be visualized in the unit-resolution importance logo (the PWM-like logo). Graphics were generated with the software seq2logo [48].

To compare interpretation method consistency across multiple sequences, we used the top-105 high affinity binding sequences (all with relative binding affinity >0.7) containing the TGATTTAT core motif from the Exd-Ubx SELEX-seq data. For each sequence $s$, each interpretability method $g$, and each hyperparameter set ${{f}_h}$ (for each model architecture, we have three sets of hyperparameter sets, noted as ${{f}_1},\ {{f}_2},\ {{f}_3}$), we derived the importance vector $g( {{{f}_h}( s )} )$. We then calculated three pair-wise Pearson correlation coefficients between the importance vectors: $c1( {g,s} ) = \textit{corr}[ {g( {{{f}_1}( s )} ),\ g( {{{f}_2}( s )} )} ],\ c2( {g,s} ) = \ \textit{corr}[ {g( {{{f}_1}( s )} ),\ g( {{{f}_2}( s )} )} ],\ and\ c3( {g,s} ) = \textit{corr}[ {g( {{{f}_2}( s )} ),\ g( {{{f}_3}( s )} )} ]$. We then calculated the average of these pair-wise correlations, denoted as $c( {g,s} ) = \textit{average}( {c1( {g,s} ),\ c2( {g,s} ),\ c3( {g,s} )} )$, for all 105 sequences and all three interpretability methods. The resulting 105 values for each interpretability method is plotted in a violin plot, and the same analysis was also performed using Spearman’s rank correlation.

### Pyrimidine-purine (YR) base-pair encoding for aligned high- and low-affinity TFBSs

To visualize high- and low-affinity TFBSs highlighted by the sequence-based CNN models, 14-mer SELEX-seq data for Exd-Ubx were first aligned. In each raw DNA sequence, each motif scanner highlighted a motif that had the largest value at the convolution layer. Because different motif scanners derived different sets of aligned motifs, only the one with the largest information content was used for visualization. Here, the information content was calculated using the RSAT matrix-clustering tool [49]. The sequence-based CNN model used here was CNN-RC-augmented with its best-performing hyperparameter setting. After alignment, aligned motifs in sequences with the highest 10 000 and lowest 10 000 binding affinities were used for visualization. Both the PWMs and YR logos were plotted with WebLogo 3 [50].

## Results

### The CNN and SA models outperform the MLR model in quantifying a large spectrum of binding affinities

To investigate differences between traditional statistical machine learning methods and CNN-based/SA-based models in the modeling of *in vitro* TF–DNA binding affinity, we trained all models using the raw SELEX-seq data of eight Exd-Hox heterodimers: Exd-Lab, Exd-Pb, Exd-Dfd, Exd-Scr, Exd-Antp, Exd-Ubx, Exd-AbdA, and Exd-AbdB (see the “Materials and methods” section; Supplementary Table S1). These Exd-Hox TF heterodimers regulate the segment-specific development of the *Drosophila* embryo along its anterior-posterior axis. The datasets contain DNA sequences with a large spectrum of relative binding affinities. Different binding mechanisms of Exd-Hox protein complexes have been previously discovered through quantitative modeling and experimental validation. For instance, the high-affinity binding sites of these Exd-Hox proteins are three closely related sequences: Exd-Lab and Exd-Pb prefer TGATTGAT, Exd-Dfd and Exd-Scr prefer TGATTAAT, and Exd-Antp, Exd-Ubx, Exd-AbdA, and Exd-AbdB prefer TGATTTAT [13]. Meanwhile, homotypic low-affinity binding sites at the *svb* enhancer were found to contribute to the binding specificity of Exd-Ubx [42]. These data provided a valuable resource to evaluate the ability of quantitative models to unravel binding mechanisms.

**Figure 2.**
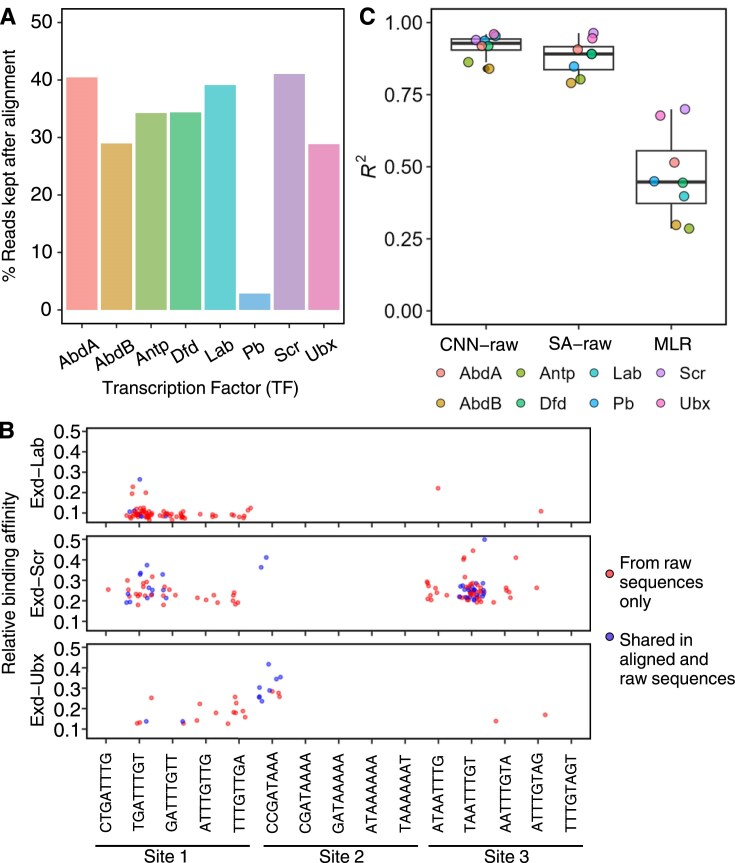
The CNN and SA models outperform the MLR model. (**A**) Bar plot showing percentage of reads retained after the read alignment step required by MLR models. (**B**) Abundance of low-affinity site subsequences in original SELEX-seq and aligned dataset. Each dot represents one match of the subsequence on the *x*-axis to one sequence in the SELEX-seq data. The central line shows the median, the box spans the interquartile range, and the whiskers extend to data points within 1.5 × IQR from the quartiles (with individual points beyond shown as outliers). (**C**) Box plots comparing model performances of the MLR and CNN/SA models.

We first trained an MLR model with L2 regularization (ridge penalty) using this dataset. The MLR model assigns weights specific to nucleotide positions; thus, it requires an alignment step. The alignment step required for the MLR model largely reduced the number of sequences that could be used in training, resulting in fewer than 42% of raw sequences in the SELEX-seq dataset (Fig. 2A and Supplementary Table S1). We further investigated the alignment step using the three low-affinity binding sites on the *svb* enhancer (Supplementary Fig. S2): CTGATTTGTTGA (Site 1), CCGATAAAAAAT (Site 2), and ATAATTTGTAGT (Site 3). We searched for the occurrence of all 8-mer subsequences (i.e. for Site 1, we searched for CTGATTTG, TGATTTGT, GATTTGT, ATTTGTTG, and TTTGTTGA) in the SELEX-seq dataset. We found that more than half of the SELEX-seq reads that contained a match were dropped (Fig. 2B and Supplementary Figs S3  and S4).

In contrast, the CNN and SA models can process raw data without requiring sequence alignment due to their architectural advances (i.e. convolutional layer and position embedding with the attention mechanism). We carefully trained a CNN model and an SA model using the entire SELEX-seq raw dataset, namely CNN-raw and SA-raw (loss curve shown in Supplementary Figs S5 and S6). The CNN and SA models significantly outperformed the MLR model in modeling the relative binding affinities of all eight Exd-Hox heterodimers (Fig. 2C). Despite similar distribution of relative binding affinity before and after the sequence alignment step (Supplementary Fig. S7), to control for the effect of training sample difference, we also trained CNN-raw and SA-raw on the same dataset as used for the MLR model (models named CNN-aligned and SA-aligned). Both the CNN and SA models outperform MLR (Supplementary Fig. S8 and  Supplementary Table S5).

Additionally, we trained MLR, CNN-raw, and SA-raw on the TDC-aligned dataset that was aligned using Top-Down Crawl [23] with 98% of the raw data considered (see the “Materials and methods” section) and observed the same result (Supplementary Table S5).

### Strategies of input data augmentation affect model performance

Owing to their double-stranded nature, DNA sequences are a special type of input data that have two-directional (5′ to 3′) strands of sequence representing the same molecule, which poses a challenge for model design. Ideally, a biologically relevant model should generate the same output for an input DNA sequence and its reverse-complement sequence. One solution is to design models with a special architecture that can accommodate this characteristic, enforcing the same output for any sequence and its reverse-complement sequence. Another solution is to apply DNA-specific input data augmentation strategies.

To systematically evaluate the effect of input data augmentation strategies on model performance and model consistency, we first trained CNN-RC [45], a CNN-based model with a special architecture design to enforce identical outputs for the forward and reverse-complement strands (Supplementary Fig. S1). Next, we trained three CNN models with the same architecture using three input augmentation strategies. CNN-raw was trained with the raw SELEX-seq dataset. For CNN-double, the reverse-complement sequence was added to the training dataset, with the relative affinity value being identical to the original sequence in the SELEX-seq data. The RC-augmented strategy used extended sequences as input, where the reverse-complement sequence was appended to the original sequence, with Ns padded in between (Fig. 1A). We also trained three SA models with these data augmentation strategies: SA-raw, SA-RC-augmented, and SA-double.

**Figure 3.**
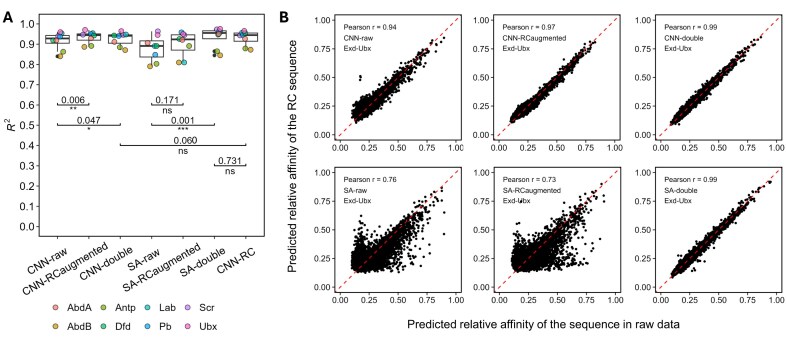
Comparison of performance achieved by seven models. (**A**) Box plots showing the performance of all models in predicting binding affinity for eight Exd-Hox complexes using SELEX-seq datasets. *P*-values of one-sided paired t-tests are shown in the plot (with the alternative hypothesis that the model on the left side achieves less accuracy than the model on the right side). The central line shows the median, the box spans the interquartile range, and the whiskers extend to data points within 1.5× IQR from the quartiles (with individual points beyond shown as outliers). (**B**) Comparisons of predicted relative affinity for given sequences and corresponding reverse-complement sequences with CNN models (upper row) or SA models (lower row) trained with raw data (left column), using “RC-augmented” as the input data strategy [middle column, with *y*-axis showing the predicted relative binding affinities of RCaug(RC(sequence))], and using “double” as the input data strategy (right column).

All seven models achieved high accuracies in predicting the relative binding affinity of Exd-Hox heterodimers (Fig. 3A). For the data input augmentation strategies, the CNN-double and CNN-RC-augmented models outperformed CNN-raw; SA-RC-augmented performed similarly to SA-raw; SA-double performed better than SA-raw; and both CNN-double and SA-double showed comparable performance to CNN-RC (Fig. 3A and Supplementary Table S5). Albeit statistically significant, the data augmentation strategies provide only limited accuracy improvement. Next, we evaluated the consensus of each model in predicting the relative binding affinity for the test data sequence and the corresponding reverse-complement sequence. By design, CNN-RC and models using the RC-augmented strategy will output the same value for the constructed sequence, the test data, and its reverse complement sequence (Supplementary Fig. S9). When comparing model architectures, we observed that CNN-raw gave a more consistent prediction than SA-raw (Fig. 3B). Moreover, CNN-double and SA-double both showed improved agreement when compared to CNN-raw and SA-raw (Fig. 3B), indicating that the “double” data augmentation strategy can be helpful when training general-purpose DNA deep learning models without a special design to handle reverse complementarity. For the RC-augmented strategy, we also evaluated an alternative approach of constructing the input sequence to the model. We applied the RC-augmented operation on the reverse-complemented sequence of the raw data. We observed that SA-double shows improved consistency (Fig. 3B).

Overall, CNN-RC showed the best model consistency and highest performance. Therefore, to further study model interpretability methods and the binding specificity of low-affinity TFBSs, we chose the CNN-RC model as a benchmark and the SA-double model as a complement.

### ISM is less sensitive to hyperparameters and model architecture

After obtaining models with good performance, it is indispensable to interpret these models and extract important information that can further provide biological insights. We investigated four interpretation methods that aim to calculate a unit-resolution importance matrix, where positive values in the matrix indicate that a small change to a nucleotide at a certain position will increase the predicted binding affinity (Fig. 1C).

**Figure 4.**
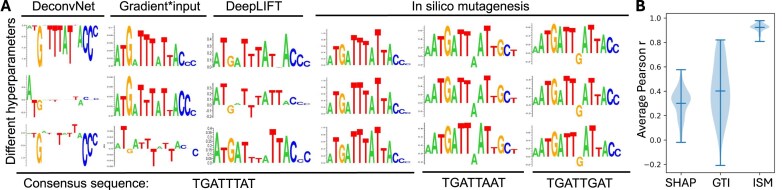
Comparison of four interpretation methods. (**A**) Models with similar performance (*R*^2^ ranges from 0.980 to 0.985) but with slightly different hyperparameter configurations were used to interpret three high-affinity TFBSs for Exd-Hox heterodimers. DeconvNet, Gradient*input, and *in silico* mutagenesis (ISM) were implemented with CNN-RC models based on Exd-Ubx SELEX-seq data. DeepLIFT was implemented with CNN-double because the DeepLIFT implementation is currently not compatible with CNN-RC models. Each row represents a specific hyperparameter configuration during model training. (**B**) Violin plot showing the comparison of consistency between different model interpretation methods (SHAP, Gradient*input (GTI), and ISM), as measured by the average pairwise Pearson correlation coefficient between the importance scores derived from models trained with different hyperparameters, across 105 different sequences.

First, we investigated the sensitivity of these interpretation methods to changes in hyperparameter settings. This choice was motivated by the observation that different combinations of learning rates could result in models with comparable performance (Supplementary Tables S6 and S7). Due to random factors (e.g. dropout techniques used during training), models trained with different sets of hyperparameters will probably stand out in performance and be selected for interpretation in different experiments. To provide valid interpretation results, an interpretation method is supposed to show a similar unit-resolution importance matrix for a given sequence while interpreting models of similar performance. We investigated the robustness of different interpretation methods with CNN-RC models built using Exd-Ubx SELEX-seq data. The sequence used to visualize the unit-resolution importance matrix was TGATTTAT (high-affinity TFBS for Exd-Ubx). As anticipated, interpretation methods mostly displayed positive and high importance scores for each nucleotide in TGATTTAT (Fig. 4). However, while interpreting TGATTTAT with models based on different hyperparameters, DeconvNet [30], Gradient*input [31], and DeepLIFT [31] occasionally showed negative weights for the TG or TA bp steps. In contrast, ISM outputs highlighted TGATTTAT consistently (Fig. 4). Although DeconvNet and Gradient*input have been successfully applied to image classification problems, which have continuous data as input, they might not be suitable for gradient-based backpropagation methods to handle the sparse data type of one-hot encoding DNA sequences. In a sparse space, taking gradients requires more caution than in a continuous space because a minor distortion in input in different directions might result in very different changes in output. We observed similar results when we applied ISM, Gradient*input, and SHAP to SA-raw; namely, ISM is the least sensitive to different hyperparameters used during model training (Supplementary Fig. S10). To test if this observation is generalizable to different sequences, we quantified the similarity of importance matrices derived from models trained with different hyperparameter sets and evaluated this similarity across a large set of sequences (see the “Materials and methods” section). We also observed that ISM gives the most consistent importance matrices (Fig. 4B and Supplementary Fig. S11).

### Recovery of functional Exd-Ubx binding at low-affinity binding sites

A widespread assumption is that TFs bind to high-affinity sequences with the lowest free energy that is most favorable for binding. Studies almost always use known cognate TFBSs or PWMs to infer both *in vitro* and *in vivo* TFBSs. Yet recent evidence suggests that TFs bind to TFBSs with a spectrum of binding affinities both *in vitro* and *in vivo* [42, 51–54]. Crocker *et al.* showed that Exd-Ubx binds specifically to three low-affinity sites on the *svb* enhancer [42]. Moreover, Crocker *et al.* found that replacing these low-affinity sites with high-affinity sequences led to non-specific binding of other Hox TFs and caused a phenotypic change in *Drosophila melanogaster* [42]. In this study, we trained deep learning models that used not only sequences containing cognate TFBSs but also those with lower affinities. Next, we evaluated whether our deep learning models and model interpretation method could recapitulate known characteristics of functionally important low-affinity binding sites used by Exd-Ubx to achieve specificity.

**Figure 5.**
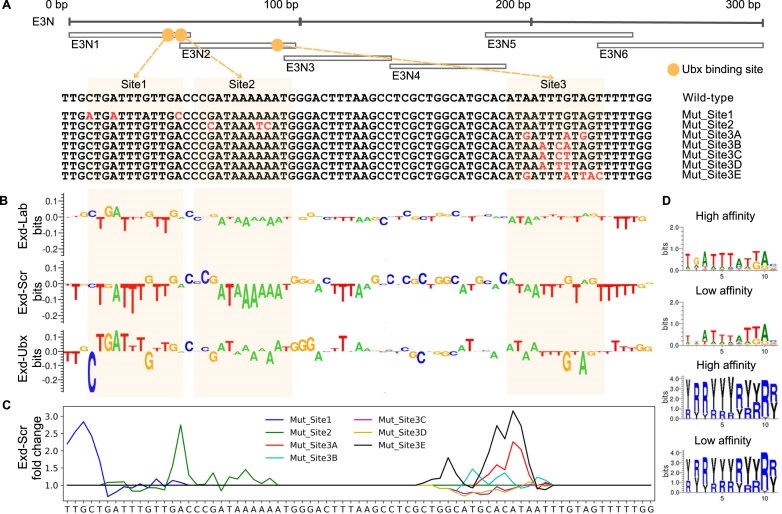
Recovery of binding specificity of Exd-Ubx to *svb* enhancer. (**A**) Illustration of three low-affinity Ubx binding sites on *svb* enhancer adapted from Crocker *et al.* [42]. (**B**) Unit-resolution importance matrix of the 74-bp WT sequence of *svb* enhancer, based on CNN-RC models trained with Exd-Lab, Exd-Scr, and Exd-Ubx SELEX-seq data, using ISM as model interpretation method. (**C**) Line plots illustrate fold change of predicted relative binding affinity of Exd-Scr due to mutations introduced to WT *svb* enhancer. Mutated sequences are as shown in panel (A). (**D**) PWM and YR (R = A or G; Y = C or T) logos for high- and low-affinity TFBSs of Exd-Ubx heterodimer.

We first applied CNN-RC models for Exd-Ubx, Exd-Scr, and Exd-Lab to the 74-bp wild-type (WT) *svb* enhancer sequence (Fig. 5A) and performed ISM interpretation to obtain the unit-resolution contribution scores. In general, we observed that a larger number of nucleotides were disfavored by Exd-Lab and Exd-Scr than by Exd-Ubx. Most nucleotides in Sites 1–3 contributed negatively to Exd-Scr binding. In addition, even though Sites 1–3 were low-affinity TFBSs for Exd-Hox heterodimers, the WT sequence showed the most positive contribution to Exd-Ubx when compared to other heterodimers, especially on Site 1 (Fig. 5B).

Next, we asked whether our CNN-RC model could recapitulate the reduced binding specificity of Exd-Ubx on the mutated sequences. In Crocker *et al.*, one striking observation was that when low-affinity Sites 1–3 were replaced by higher-affinity TFBSs, the *svb* enhancer was also recognized by other Exd-Hox heterodimers [42]. For example, Exd-Scr started to bind when the relative binding affinity increased above around 0.25, according to the embryo images (Fig. 4 of Crocker *et al.* [42]). When we applied our CNN-RC model of Exd-Ubx, we observed increased binding affinity across the *svb* enhancer for all seven mutations (Supplementary Fig. S12). In accordance with the findings of Crocker *et al.* [42], we likewise observed an increased relative binding affinity of Exd-Scr on the mutated sequences (Fig. 5C). We observed the same results when we performed these predictions with the SA-double models for Exd-Ubx and Exd-Scr (Supplementary Fig. S13).

After recovering the *in vivo* phenotypic evidence, we examined the possible mechanism behind the binding specificity of Exd-Ubx at low-affinity binding sites. Three classes of high-affinity TFBSs (TGATTGAT for Exd-Lab, TGATTAAT for Exd-Scr, and TGATTTAT for Exd-Ubx) only differed at the underlined nucleotide position, and Exd-Ubx specifically favored a pyrimidine at that position. It has been shown that pyrimidine (Y)–purine (R) stacking at YR bp steps is particularly weak compared to other bp steps and can cause cross-strand steric clashes [55], which can reduce local DNA stability [56]. Moreover, fluctuating flexibility in the local helical conformation of DNA can lead to infrequent events of bp opening, thus exposing buried groups for interactions with proteins [57]. After encoding high-affinity TFBSs of Exd-Hox heterodimers into YR representations, we found that only Exd-Ubx had two YR bp steps (underlined in YRRYYYRY) compared to Exd-Scr and Exd-Lab. To examine whether YR stacking can explain why Exd-Ubx binds specifically to low-affinity TFBSs, we used thoroughly trained CNN models to align SELEX-seq probes (see the “Materials and methods” section), selected 10 000 probes each with lowest and highest binding affinities, and visualized these probes in YR representations. Although the DNA sequence preference at position 2 was different between high- and low-affinity TFBSs, the preference for purine versus pyrimidine identities was always YRRYYYRY for Exd-Ubx (Fig. 5D). Based on these observations, we suggest that YR stacking might be a driving force for Exd-Ubx to bind to DNA, whereas the functional groups of DNA nucleotides fine-tune high-affinity TFBSs and differentiate them from low-affinity TFBSs.

## Discussion

TF–DNA binding specificity is affected by many molecular determinants, such as DNA sequence and shape [43, 58], cofactors and cooperative binding [13, 59, 60], and epigenetic marks [11, 61, 62]. Deep learning models with different architectures have improved the accuracy of modeling TF–DNA binding specificity and provided effective solutions to limitations in traditional motif discovery [32, 35–37, 63–67]. These models are usually alignment-free methods that can capture non-linear dependencies between TFBS nucleotides. However, interpretability is challenging compared to traditional machine learning methods such as MLR or boosting. The contribution of nucleotide importance at each nucleotide position within the TFBS can be derived by gradient-based backpropagation methods [29, 30], DeepLIFT [31], or ISM [32, 33]. Still, we faced two problems while applying sequence-based deep learning models in understanding the binding specificity of Exd-Hox heterodimers in *Drosophila*. The first challenge was how to handle the orientation of DNA sequences, and the second was the choice of interpretation method.

To solve the first problem, we took advantage of high-quality SELEX-seq data for eight Exd-Hox heterodimers from *Drosophila* that covered a large spectrum of binding affinity values. These homologous TFs have been well-documented to utilize varied binding mechanisms, such as the interplay of DNA base and shape readout [43], cofactors [13], and the occupancy of clusters of low-affinity TFBSs [42]. We first trained four sequence-based CNN models: CNN-raw, CNN-RC-augmented, CNN-double, and CNN-RC. The first three used the same underlying CNN model, but their input was constructed differently (see the “Materials and methods” section). CNN-RC used the same input as CNN-raw but had a special weight-sharing layer that forced a sequence and its reverse-complement sequence to be treated the same. In the results, the CNN-RC model had higher consistency in terms of providing identical predicted binding affinity for the two sequence orientations. CNN-RC had a minor drawback of needing additional preprocessing to obtain the orientation in which a TF really binds, whereas the canonical models could output the orientation with a larger predicted binding affinity. In addition, we trained three SA-based models: SA-raw, SA-double, and SA-RC-augmented (SA-RC was not implemented due to the special implementation of CNN-RC). SA-double performed similarly to CNN-RC and provided insight into how attention mechanisms can be used in predicting TF–DNA binding.

To solve the second problem, we evaluated four network interpretation methods that are widely used in image classification and applications in DNA sequence analysis: Gradient*input [31], DeconvNet [30], DeepLIFT [31], and ISM. After obtaining unit-resolution importance matrices for high-affinity TFBSs of Exd-Ubx, we found that the ISM method was the least sensitive to different model training hyperparameters. While there can be an argument made that models trained with different hyperparameters can learn distinct internal representations and some variation in model interpretation is expected, the aim of this work was to provide another possible perspective—if interpretation methods can uncover consistent meaningful biological signals.

We also recovered the *in vivo* binding specificity of Exd-Ubx on the *svb* enhancer, which can be beneficial for the future design of potential TFBSs *in vivo*. We stress that the sequence-based CNN models were built with 14-mer SELEX-seq data, which are assumed to have one TFBS in each 14-mer. While interpreting *in vivo* DNA sequences containing multiple TFBSs due to cooperativity, mutation of one TFBS could be compensated by other TFBSs and does not necessarily reduce binding. In this case, other interpretation methods might be applicable. We also visualized attention maps using the SA model for Ubx with the WT and mutated sequences on the *svb* enhancer. We observed similar patterns for high-affinity sequences that contained a TGATTTAT site. In contrast, the low-affinity sequence tended to have more spread-out SA (Supplementary Fig. S14). However, the attention maps are sequence-specific, and these observations may not be extended as a general conclusion for all sequences. Finally, visualizing attention maps is not directly comparable to the model interpretation methods that we studied. SA scores only measure interaction and do not indicate feature importance such as methods like ISM. Nevertheless, SA design can be very useful for studying long-range interactions and cooperativity between TFs, especially with datasets consisting of long genomic sequences and spaced motifs for multiple TFs.

Our approach has several limitations. One limitation is that we performed all our analyses only on datasets from eight homeodomain SELEX-seq experiments. These data do not represent all TF families and/or all experimental platforms. Different TFs may have different DNA readout mechanisms, and the model that performs best for homeodomains may not perform well for another TF family. Different experimental approaches may generate data with different biases or variance and have different sensitivities. The best hyperparameter can be dramatically different for different data. The second limitation is that the sequence length in the dataset was only 14 bp, which is not an ideal length for SA models. The sequence length also eliminates the need for a deeper CNN model, as a suitable filter length and shallower model are sufficient to locate the motif and predict the relative binding affinity.

In summary, we proposed and identified potential solutions to two problems in quantifying TF–DNA binding specificity: a strategy to handle sequence orientation and a proper network interpretation method. Taking advantage of SELEX-seq data for eight Exd-Hox heterodimers and *in vivo* binding evidence on the *svb* enhancer in *Drosophila*, we found that the CNN-RC model, combined with the ISM interpretation method, is a good combination to investigate TF–DNA binding specificity. The method can be used to study low-affinity TF binding sites.

## Supplementary Material

lqag027_Supplemental_File

## Data Availability

The 14-mer dataset of eight *Drosophila* SELEX-seq experiments and source code for implementing data preprocessing, L2-MLR models, sequence-based CNN models, SA models, and deriving the unit-resolution importance matrix are available in a GitHub repository at https://github.com/wangyingfei/cnn-sa-lowaffinity-tfbind and through figshare at https://doi.org/10.6084/m9.figshare.28792001.v1.
