# Supplementary material for: Sequence-based modeling of low-affinity transcription factor–DNA binding through deep learning
Source: NAR Genom Bioinform. 2026 Mar 5;8(1):lqag027. doi: 10.1093/nargab/lqag027 (PMC12961433; doi:10.1093/nargab/lqag027)
Supplement: lqag027_Supplemental_File [file lqag027_supplemental_file.pdf]

SUPPLEMENTARY DATA

Sequence-based modeling of low-affinity transcription factor–DNA binding through deep learning

Yingfei Wang[1,†], Jinsen Li[1,†], Tsu-Pei Chiu[1], Beibei Xin[2,*], and Remo Rohs[1,3,4,5,6,7,*]

[1]Department of Quantitative and Computational Biology, University of Southern California, Los Angeles, CA 90089, USA

[2]State Key Laboratory of Maize Bio-Breeding, Department of Plant Genetics and Breeding, China Agricultural University, Beijing 100193, China

[3]Department of Chemistry, University of Southern California, Los Angeles, CA 90089, USA

[4]Department of Physics & Astronomy, University of Southern California, Los Angeles, CA 90089, USA

[5]Thomas Lord Department of Computer Science, University of Southern California, Los Angeles, CA 90089, USA

[6]Division of Medical Oncology, Department of Medicine, University of Southern California, Los Angeles, CA 90033, USA

[7]Alfred E. Mann Department of Biomedical Engineering, University of Southern California, Los Angeles, CA 90089, USA

*To whom correspondence should be addressed. Email: rohs@usc.edu

Correspondence may also be addressed to Beibei Xin. Email: bxin@cau.edu.cn

[†]The first two authors should be regarded as Joint First Authors.

SUPPLEMENTARY FIGURES

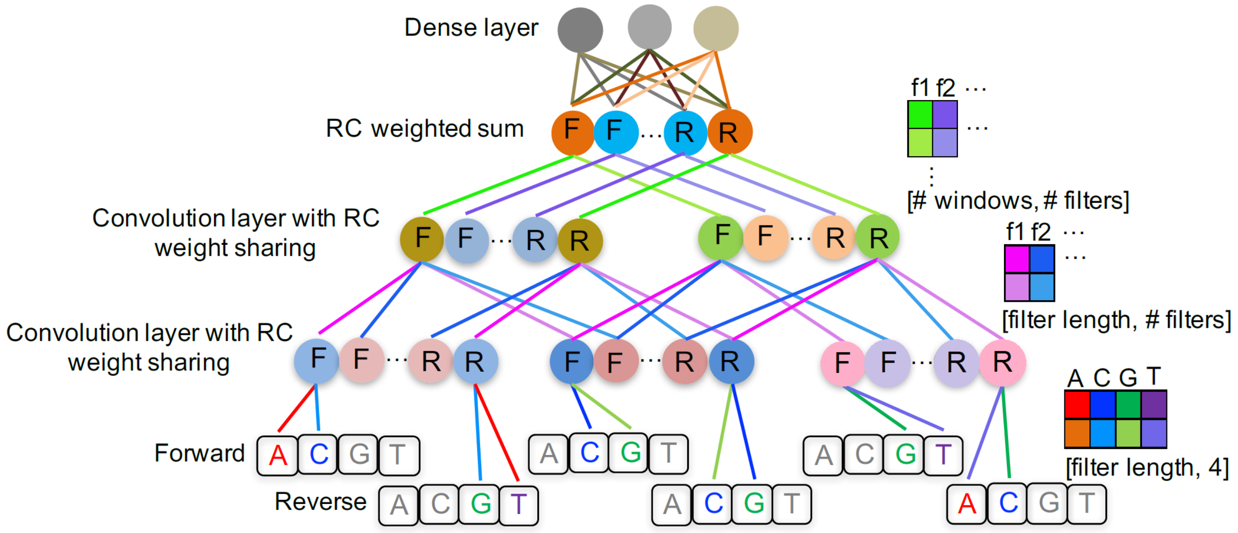

Supplementary Figure S1. Demonstration of architecture of CNN-based reverse-complement (RC) weight-sharing models.
Input sequence $s = \text{'ACGT'}$. Width of each scanner is $m = 2$, also known as filter length. Weights of different values at each layer are marked by different colors, as indicated in each color table on the right side of the network. Edges on the network have colors matched to corresponding weights in the color table. Different colors of neurons represent different values. ‘F’ and ‘R’ on each neuron mean ‘Forward strand’ and ‘RC strand’, respectively. In the first convolutional layer, obtaining the reverse complement (RC) of a filter involves reversing its weights along the sequence length and swapping the A↔T and C↔G channels.

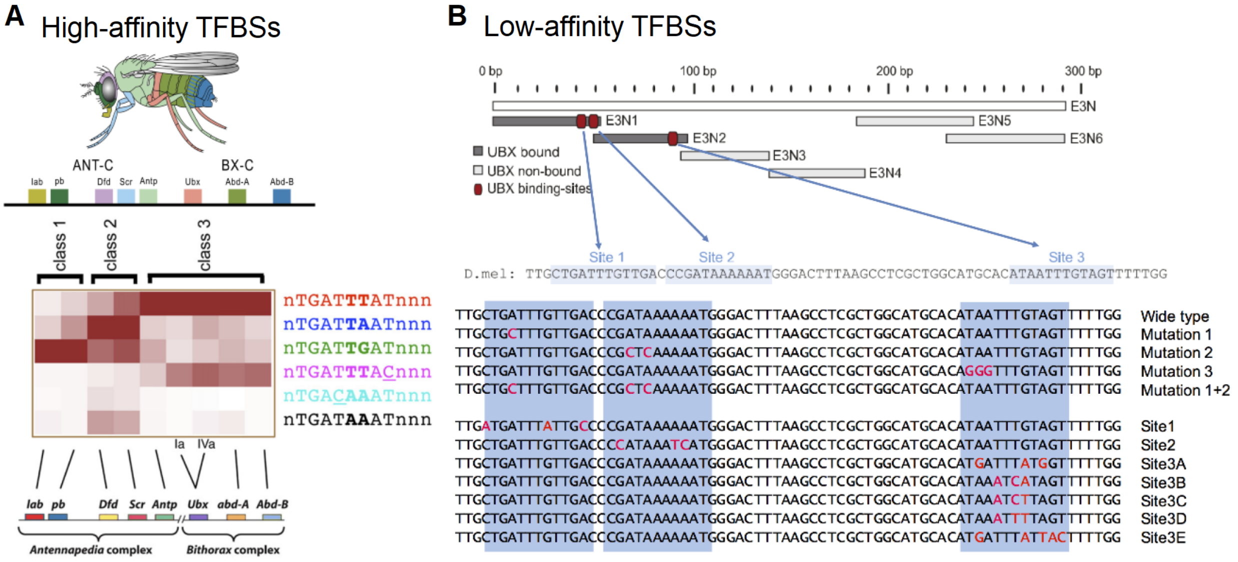

Supplementary Figure S2. High- and low-affinity TFBSs in genome of a *Drosophila* embryo. (A) Schematic figure showing eight Exd-Hox heterodimers that control body plan of *Drosophila* embryo along anterior-posterior axis (adapted from https://en.wikipedia.org/wiki/Hox_gene). Heat map shows three classes of Exd-Hox heterodimers. Each class prefers a slightly different consensus sequence TGAYNNAY (adapted from Slattery et al. (1)). (B) Three low-affinity TFBSs on *svb* enhancer were identified for Exd-Ubx binding *in vivo*. Four different mutations of each of the TFBSs or seven scenarios of replacing low-affinity TFBSs with high-affinity TFBSs showed decreased binding specificity of Exd-Ubx (adapted from Crocker et al. (2)).

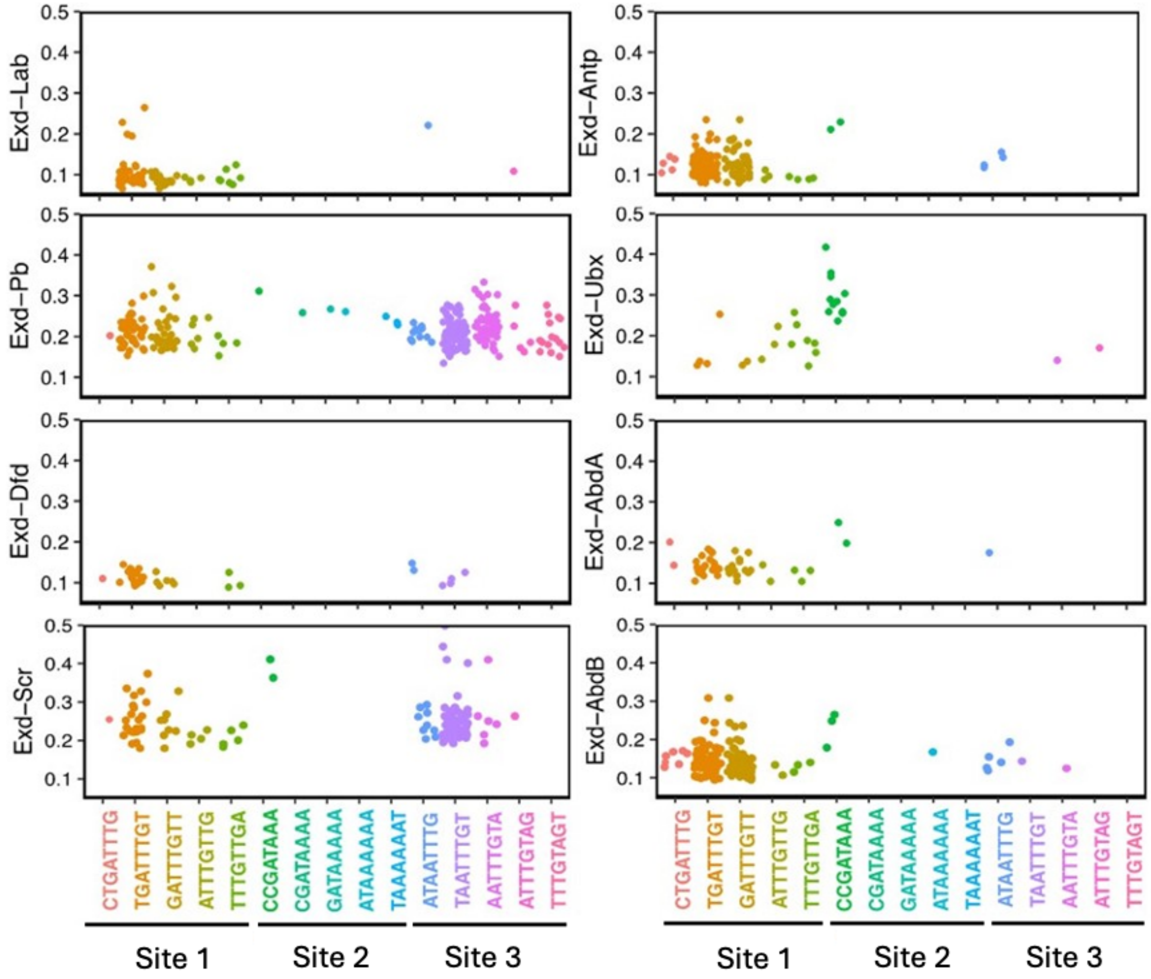

Supplementary Figure S3. Abundance of low-affinity TFBSs (Sites 1–3) reported by Crocker et al. (2) in raw 14-mer SELEX-seq data.

Jitter plots for abundance of low-affinity TFBSs in raw data. Each dot represents a raw DNA sequence in SELEX-seq data, and *y*-axis represents relative binding affinity of the sequence. Because full-length sequences of Sites 1–3 do not appear in any 14-mer, we checked the abundance of the 8-mer in each site instead.

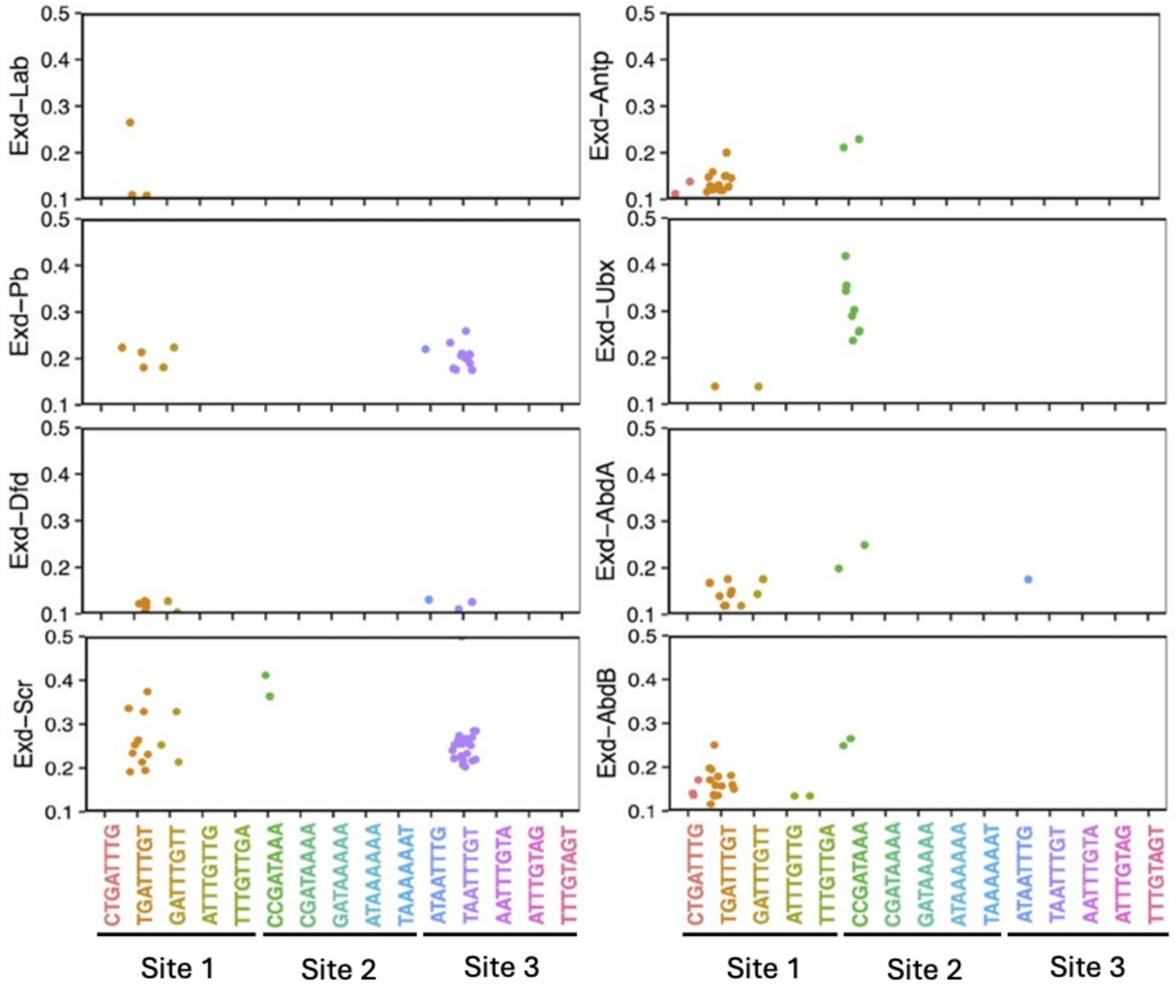

Supplementary Figure S4. Abundance of low-affinity TFBSs (Sites 1–3) reported by Crocker et al. (2) in aligned 14-mer SELEX-seq data.
Each panel shows abundance of low-affinity TFBSs in aligned sequences for each Exd-Hox heterodimer. Each dot represents a bound probe, and the *y*-axis represents relative binding affinity of TFBSs.

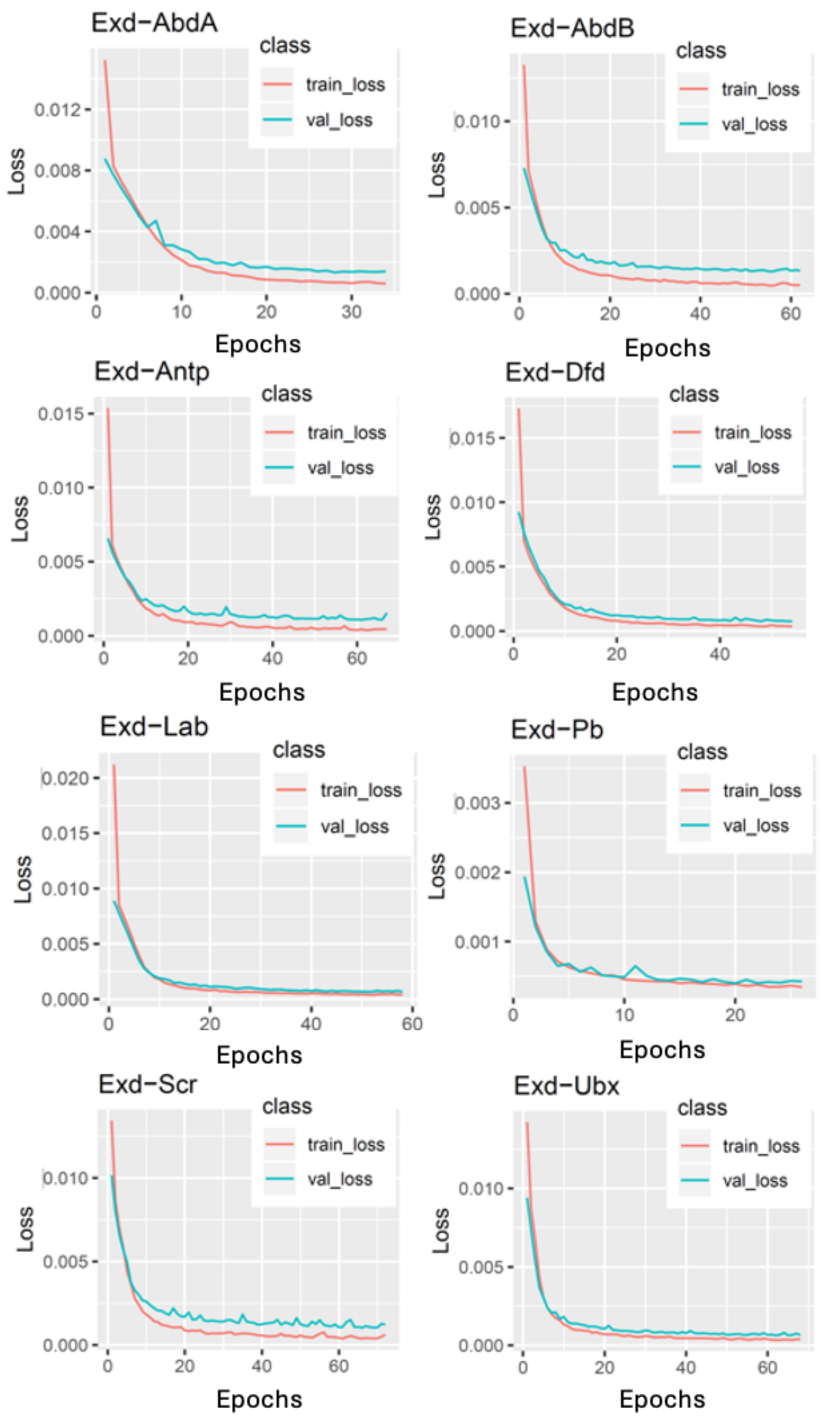

Supplementary Figure S5. Loss function during training of CNN-raw models.
Training and validation loss decrease steadily, and both are low and flattened towards end of the training.

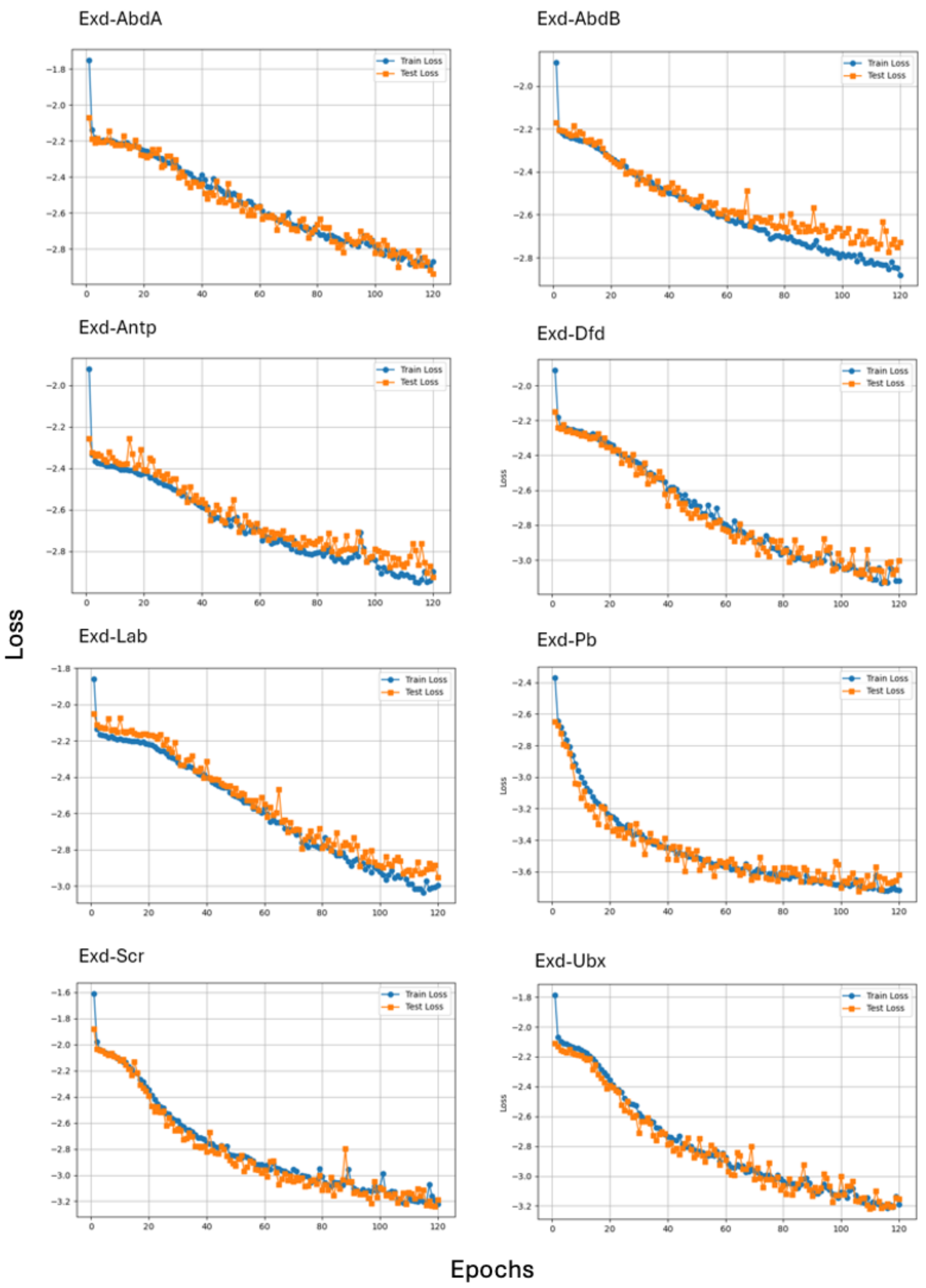

Supplementary Figure S6. Training curve during training of SA-raw models.
The *y*-axis shows *log(loss)*. Training and validation loss are both low and converging to <10^{-3} towards end of training.

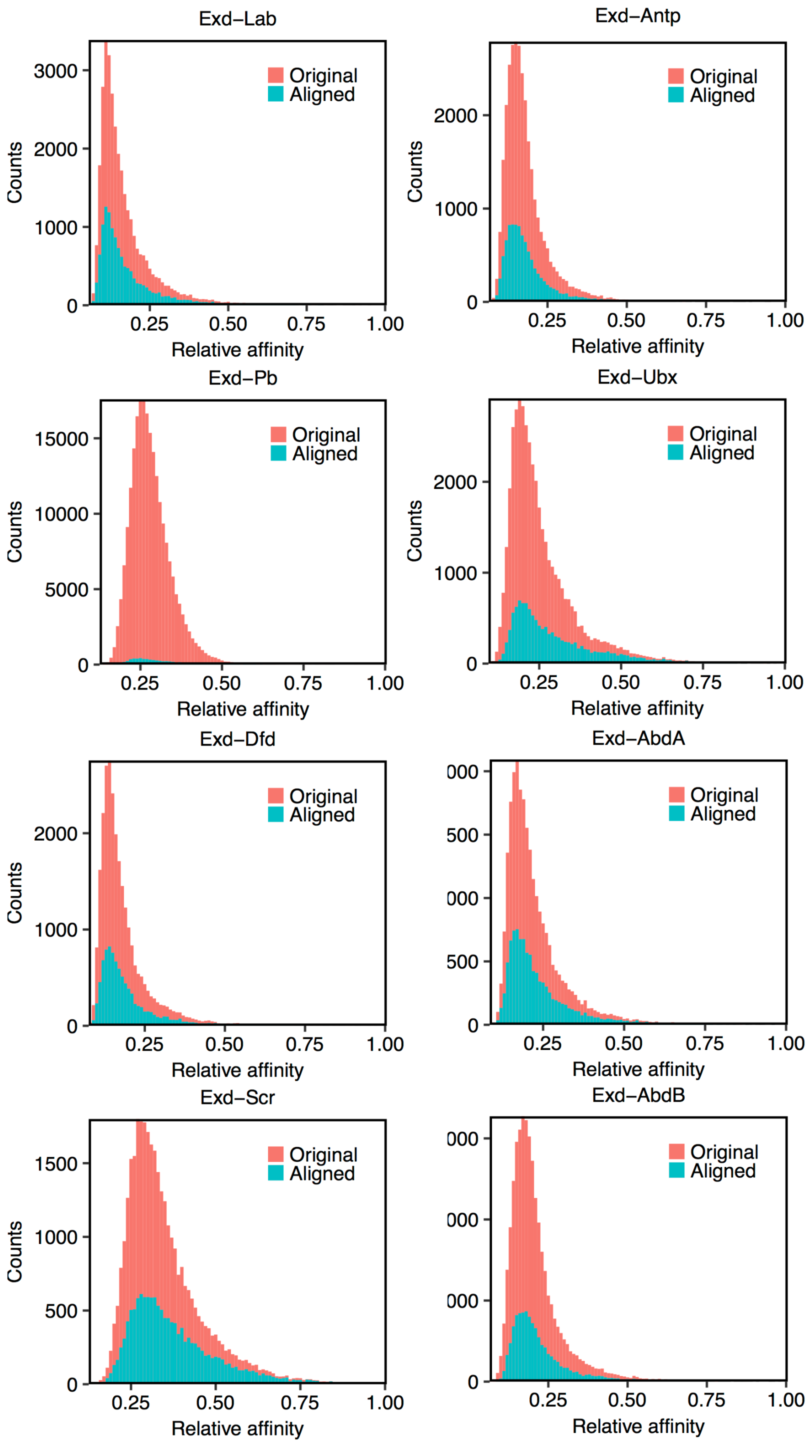

Supplementary Figure S7. Alignment to TGAYNNAY largely reduces number of samples in SELEX-seq data that are used to train MLR model.
Each panel shows distribution of binding affinities of original sequences and aligned sequences.

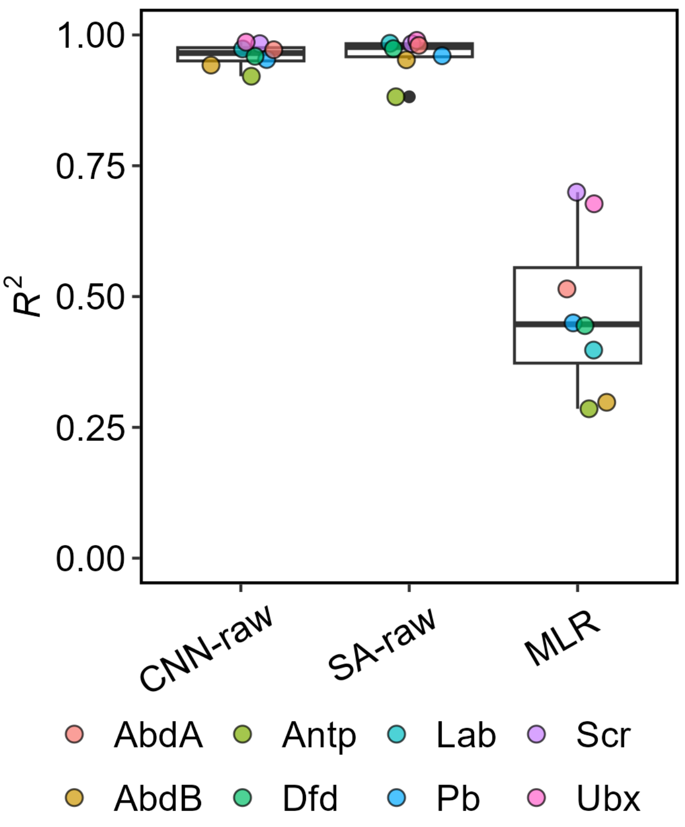

Supplementary Figure S8. Model performance of the MLR and CNN/SA model using aligned data only.

Boxplots showing model performance of MLR and CNN-raw and SA-raw models using aligned data as the training sample. The center line shows the median, the box spans the interquartile range, and the whiskers extend to data points within 1.5×IQR from the quartiles (with individual points beyond shown as outliers).

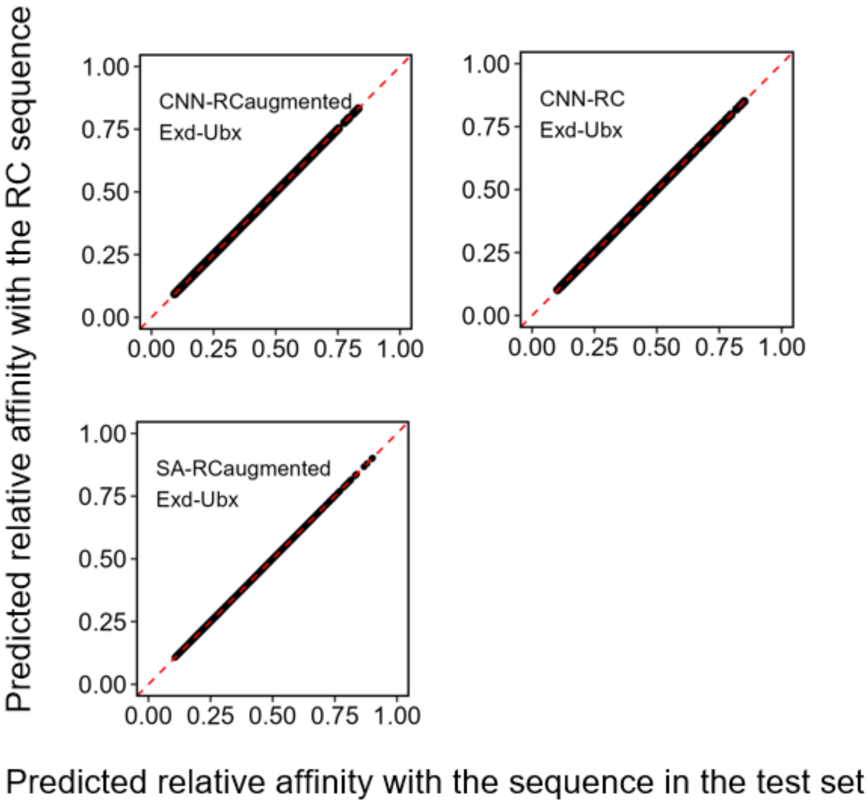

Supplementary Figure S9. **Comparison of model prediction of sequence in test set and corresponding RC sequence using CNN-RCaugmented, SA-RCaugmented, and CNN-RC models.**

Each panel shows predicted relative binding affinity for sequences in the test set and their corresponding reverse complement (RC) sequences using one of seven models. By model architecture design, CNN-RC outputs the same value for forward and RC sequences. For CNN-RCaugmented and SA-RCaugmented, the forward sequence is RCaug(raw sequence) and the RC sequence is RC(RCaug(raw sequence)). These sequences are identical, thus leading to identical prediction results.

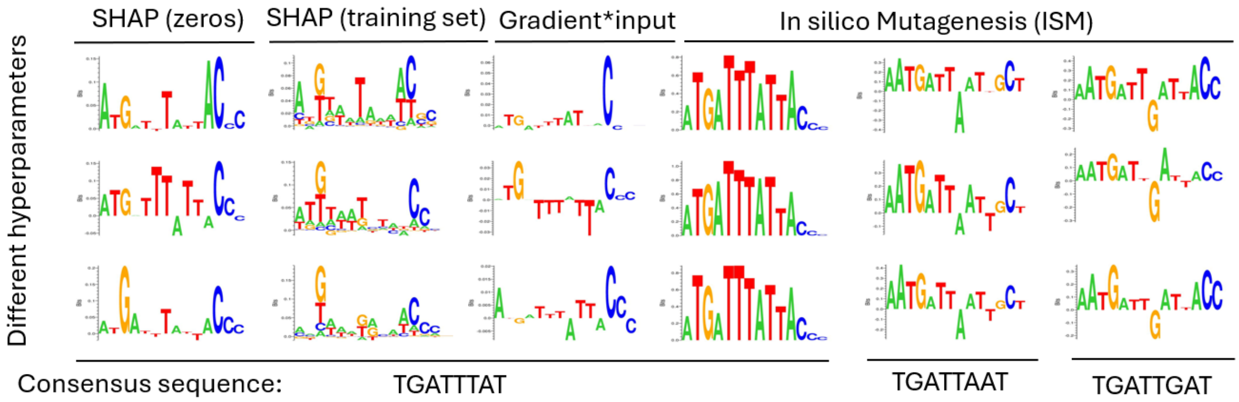

Supplementary Figure S10. Comparison of four interpretation methods on SA models. Models with similar performances but slightly different hyperparameter configurations (see Supplementary Table 6) used to interpret three high-affinity TFBSs for Exd-Hox heterodimers. All interpretation results are based on SA-raw model for Exd-Ubx. SHAP (zeros) uses all-zero vectors as background sequence. SHAP (training set) uses entire training set as background sequence. Each row represents a specific hyperparameter configuration during model training.

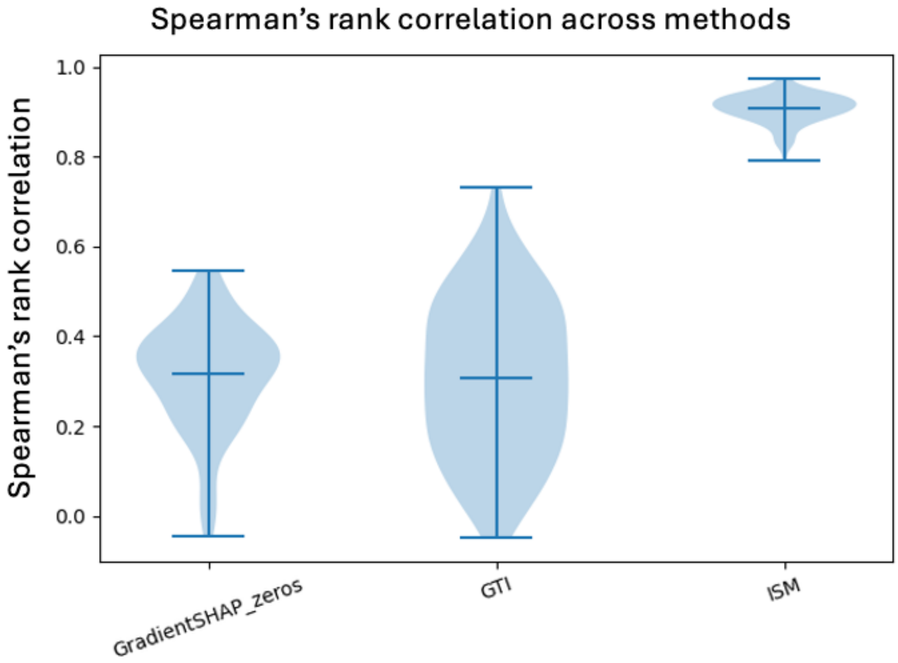

Supplementary Figure S11. Comparison of interpretation methods across top 105 high-affinity sequences containing the TGATTTAT motif.

Violin plot showing the average pairwise Spearman's rank correlation (lower panel) of the importance scores across the three SA-raw models (same architecture, trained with different training parameters) for the 105 sequences.

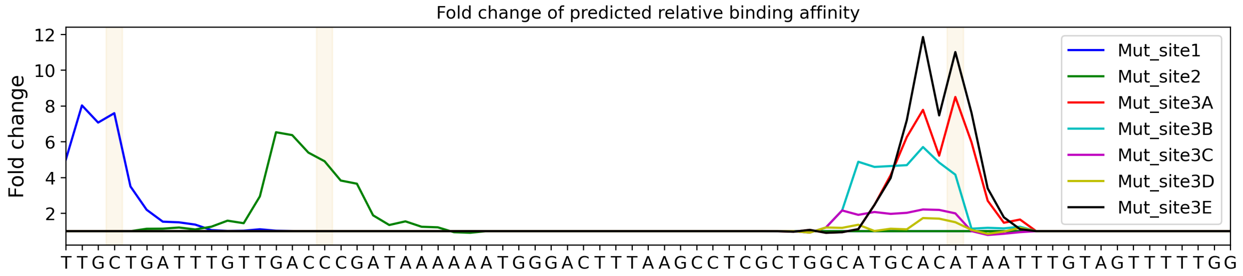

Supplementary Figure S12. CNN-RC predicts increased Exd-Ubx relative binding affinity on mutated sites on *svb* enhancer.

Fold change of predicted relative binding affinity of Exd-Ubx (relative binding affinity of mutation sequence/relative binding affinity of wild-type (WT) sequence) using CNN-RC model. We observed increased binding affinity across *svb* enhancer for all seven mutations.

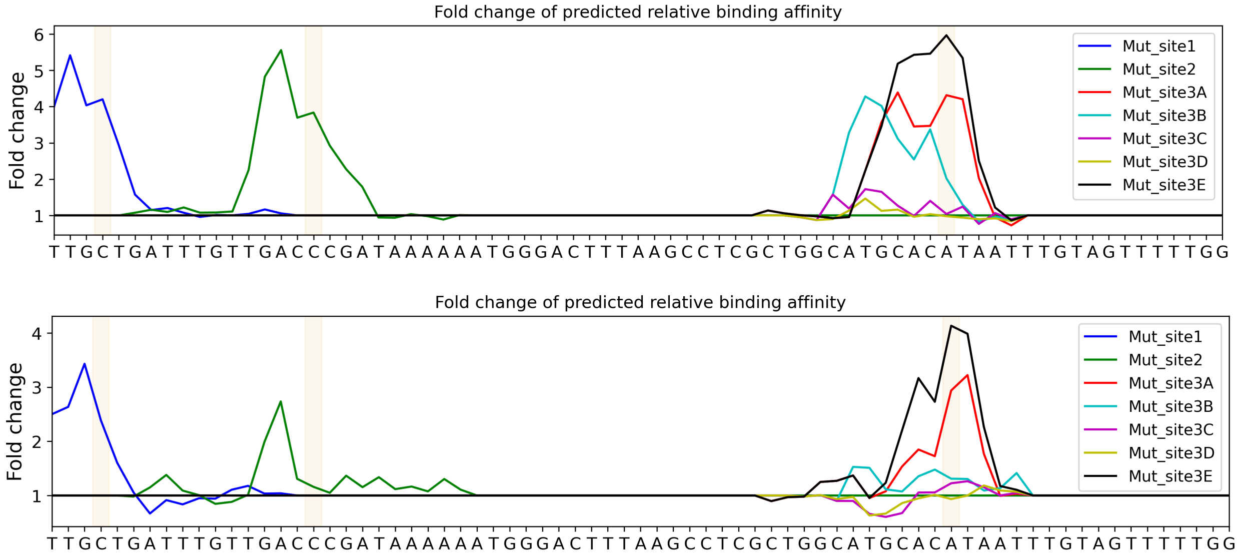

Supplementary Figure S13. SA-double predicts increased Exd-Ubx and Exd-Scr relative binding affinity on mutated sites on *svb* enhancer.

SA-double models with Exd-Ubx (upper panel) and Exd-Scr (lower panel) both predict increased binding affinities near mutated sites for all seven *svb* enhancer sequences with mutations.

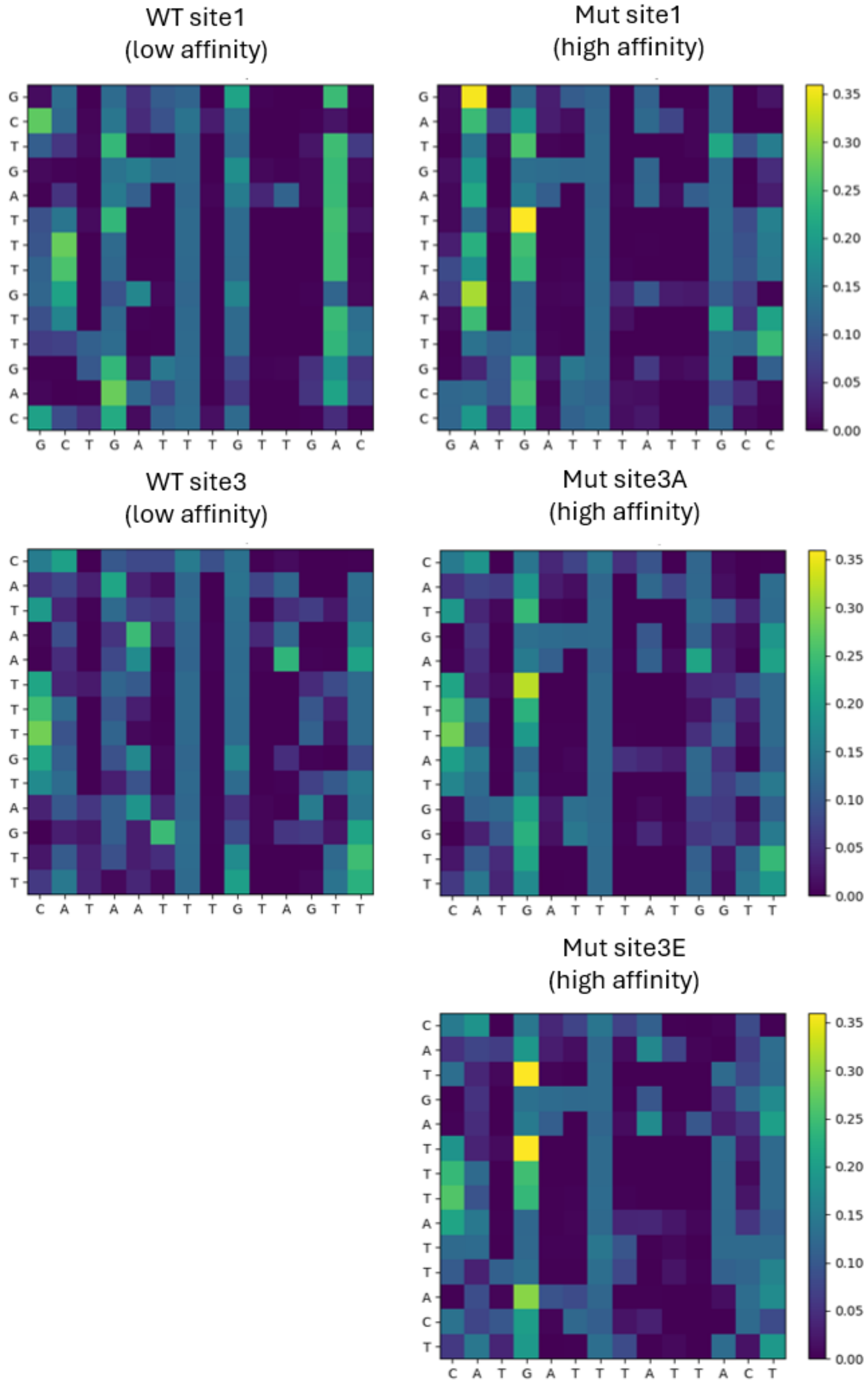

Supplementary Figure S14 Attention maps of WT sequence and three mutated sites on *svb* enhancer.

We observed similar patterns for high-affinity sequences that contained a TGATTTAT site (most-preferred sequence by Ubx), with consistent SA focusing on guanine residue in motif, and strong interaction between the guanine and the second thymine. In contrast, the low-affinity sequence tended to have more spread-out SA and did not show special focus on the guanine despite presence of a highly similar motif (TGATTTGT).

SUPPLEMENTARY TABLES

Supplementary Table S1. Sample sizes (number of DNA sequences) of eight Exd-Hox heterodimer datasets in SELEX-seq data, and number of sequences remaining after alignment to TGAYNNAY motif.

Hox protein	Lab	Pb	Dfd	Scr	Antp	Ubx	AbdA	AbdB
Sample size	32,563	228,028	26,652	34,489	30,777	42,471	25,063	41,476
Remaining data after alignment	39.11%	2.84%	34.36%	41.04%	34.24%	28.82%	40.45%	28.96%

Supplementary Table S2. Nine hyperparameter sets used for training CNN models.

LASSO weight regularization penalty at conv1 (λ_1)	Ridge weight regularization penalty at conv1 (λ_2)	Learning rate of Adam optimization (λ_3)
5e-05	5e-05	0.0001
1e-06	0.001	0.0003
0	0.001	0.0003
1e-05	0.001	0.0003
5e-06	0.001	0.0003
1e-05	1e-05	3e-05
1e-05	5e-06	5e-05
5e-06	1e-05	5e-05
1e-05	1e-05	8e-05

Supplementary Table S3. Hyperparameters used for training SA models in initial grid search (all permutations).

Learning rate	Batch size	*dmodel*	*nlayer*	*nhead*
0.001, 0.0001, 0.00001	256, 1024, 4096	128, 512	1	1, 2, 4, 8

Supplementary Table S4. Performance of SA models with different designs in self-attention layer on Exd-Ubx dataset.

nlayer	*nhead*	R^2 on test data
1	1	0.6627
1	2	0.8186
1	4	0.8867
1	8	0.9237
2	1	0.7065
2	2	0.9336
2	4	0.9431
2	8	0.9453
4	1	0.4596
4	2	0.8208
4	4	0.9186
4	8	0.9372

Supplementary Table S5. Performance of all trained models (R^2).

TF	CNN-aligned	CNN-TDC aligned	CNN-raw	CNN-RCaug	CNN-double	SA-aligned	SA-TDC aligned	SA-raw	SA-RCaug	SA-double	CNNRC	MLR-TDC aligned	MLR-aligned
Scr	0.9833	0.9540	0.9394	0.9468	0.9456	0.9831	0.9529	0.9634	0.9449	0.973	0.9433	0.7231	0.6995
Lab	0.9732	0.9348	0.9533	0.9543	0.9476	0.9840	0.9393	0.8911	0.8098	0.9598	0.9561	0.5195	0.3977
Pb	0.9530	0.8431	0.9371	0.9496	0.9402	0.9602	0.8666	0.8478	0.9585	0.9637	0.9462	0.5224	0.4496
Dfd	0.9592	0.9204	0.9187	0.9426	0.9427	0.9738	0.9190	0.8911	0.9257	0.9472	0.9547	0.4966	0.4444
Antp	0.9211	0.7959	0.8627	0.8914	0.8848	0.8818	0.8044	0.8031	0.8911	0.865	0.8788	0.2110	0.2855
Ubx	0.9862	0.9642	0.9595	0.9704	0.9645	0.9896	0.9644	0.9453	0.9501	0.9753	0.9655	0.7062	0.6773
AbdA	0.9717	0.9392	0.9192	0.9292	0.9114	0.9800	0.9460	0.9066	0.9213	0.9508	0.9264	0.5039	0.5146
AbdB	0.9424	0.7931	0.8400	0.8866	0.8708	0.9525	0.8001	0.7906	0.8097	0.845	0.8726	0.2410	0.2979

Supplementary Table S6. Performance of CNN-RC model with different hyperparameter settings on Exd-Ubx SELEX-seq data.

LASSO weight regularization penalty at conv1 (λ_1)	Ridge weight regularization penalty at conv1 (λ_2)	Learning rate of Adam optimization (λ_3)	R^2 on training set	R^2 on test set
5e-06	1e-05	5e-05	0.99251006	0.98458644
1e-06	0.001	0.0003	0.98761492	0.98014456
0	0.001	0.0003	0.98955374	0.98295927

Abbreviation: conv1=first convolutional layer.

Supplementary Table S7. Performance of SA-raw models with different training hyperparameter configurations on Exd-Ubx SELEX-seq data (three models used for Supplementary Figure 8, in corresponding order from top to bottom).

Learning rate	Batch size	R^2 on test data
0.001	256	0.9533
0.0001	256	0.9314
0.001	512	0.9460

SUPPLEMENTARY REFERENCES

1. Slattery,M., Riley,T., Liu,P., Abe,N., Gomez-Alcala,P., Dror,I., Zhou,T., Rohs,R., Honig,B., Bussemaker,H.J., *et al.* (2011) Cofactor binding evokes latent differences in DNA binding specificity between Hox proteins. *Cell*, **147**, 1270–1282.

2. Crocker,J., Abe,N., Rinaldi,L., McGregor,A.P., Frankel,N., Wang,S., Alsawadi,A., Valenti,P., Plaza,S., Payre,F., *et al.* (2015) Low affinity binding site clusters confer hox specificity and regulatory robustness. *Cell*, **160**, 191–203.
